# Climate change impacts on the distribution of the allergenic plant, common ragweed *(Ambrosia artemisiifolia)* in the eastern United States

**DOI:** 10.1371/journal.pone.0205677

**Published:** 2018-10-31

**Authors:** Michael J. Case, Kristina A. Stinson

**Affiliations:** 1 Case Research, LLC, Seattle, Washington, United States of America; 2 School of Environmental and Forest Sciences, University of Washington, Seattle, Washington, United States of America; 3 Department of Environmental Conservation, University of Massachusetts, Amherst, Massachusetts, United States of America; 4 Harvard Forest, Harvard University, Petersham, Massachusetts, United States of America; Pacific Northwest National Laboratory, UNITED STATES

## Abstract

Climate change is affecting the growth, phenology, and distribution of species across northeastern United States. In response to these changes, some species have been adversely impacted while others have benefited. One species that has benefited from climate change, historically and in response to experimental treatments, is common ragweed (*Ambrosia artemisiifolia*), a widely distributed annual weed and a leading cause of hay fever in North America. To better understand how climate change may affect the distribution of common ragweed, we built a maximum entropy (Maxent) predictive model using climate and bioclimatic data and over 700 observations across the eastern U.S. Our model performed well with an AUC score of 0.765 using four uncorrelated variables, including precipitation seasonality, mean diurnal temperature range, August precipitation, and January maximum temperature. After building and testing our model, we then projected potential future common ragweed distribution using a suite of 13 global climate models (GCMs) under two future greenhouse gas scenarios for mid and late-century. In addition to providing georeferenced hot spots of potential future expansion, we also provide a metric of confidence by evaluating the number of GCMs that agree. We show a substantial contraction of common ragweed in central Florida, southern Appalachian Mountains, and northeastern Virginia and areas of potential expansion at the northern margins of its current distribution, notably in northeastern U.S. However, the vast majority of this increase is projected to occur by mid-century and may be moderated somewhat by the 2070s, implying that common ragweed may be sensitive to climatic variability. Although other factors and modeling approaches should be explored, we offer preliminary insight into where common ragweed might be a new concern in the future. Due to the health impacts of ragweed, local weed control boards may be well advised to monitor areas of expansion and potentially increase eradication efforts.

## Introduction

Average annual temperature has increased by 1.1°C (2°F) and precipitation has increased by more than 10% over the last century in the Northeast U.S. [1]. Combined with increasing carbon dioxide (CO_2_) concentrations, these changes are already affecting species–plants are flowering earlier [2] and species’ range shifts have occurred [3,4]. For instance, in the Green Mountains, Vermont, northern hardwood trees have shifted up in elevation by nearly 100 feet between 1964 and 2004 [5]. Plant species are also moving to higher latitudes with southern species expanding into areas previously dominated by northern species in the Northeast U.S. [6]. However, not all species respond similarly to climate change [7].

One plant species that may expand its range in the Northeast U.S. is common ragweed (*Ambrosia artemisiifolia* L., Asteraceae), a widely distributed annual weed whose pollen is the leading cause of hay fever and a major trigger of asthma [8,9]. Common ragweed has strong competitive growth on frequently disturbed soils [10] and responds positively to elevated CO_2_, which can dramatically increase its growth, reproduction, and pollen output [11,12,13,14]. Once established, common ragweed forms dense monospecific stands and is well adapted to a diversity of habitats. However, ragweed generally requires full or abundant sun for germination [15] and therefore usually does not grow under full tree canopy. Consequently, it is usually found in non-forested habitats such as roadsides, abandoned fields, and agricultural croplands.

The historical distribution of common ragweed has changed with variations in climate and land use. For instance, the paleoecological records indicate that common ragweed pollen abundance increased with hot, dry climates and frequent disturbances, such as fire, grazing, and to some extent, land clearing by humans [16]. Empirical evidence also indicates that common ragweed differs in its response to temperature and growing season length in some areas compared to others [17]. In Europe, where common ragweed is an invasive non-native species, it has been increasing its distribution and is expected to further expand its range due to climate change [18,19,20,21]. Previous modeling efforts have used North American common ragweed distribution to calibrate their European models [19,20,21]; however, none have examined the potential expansion of common ragweed in North America. Moreover, we are not aware of any studies that have examined the driving climate and bioclimatic predictors of common ragweed across the heavily populated eastern U.S.

Here, we applied a maximum-entropy (Maxent) approach for modeling the current distribution of common ragweed in the eastern U.S. We used common ragweed occurrence data from the Global Biodiversity Information Facility (GBIF) and climate data from WorldClim to build and evaluate our model and identified the most important climate predictors. We then applied a suite of 13 global climate models under two future greenhouse gas scenarios to project the potential future distribution for mid and late-century and identify areas that are most susceptible to future expansion.

## Methods

### Species occurrence data

**Fig 1:**
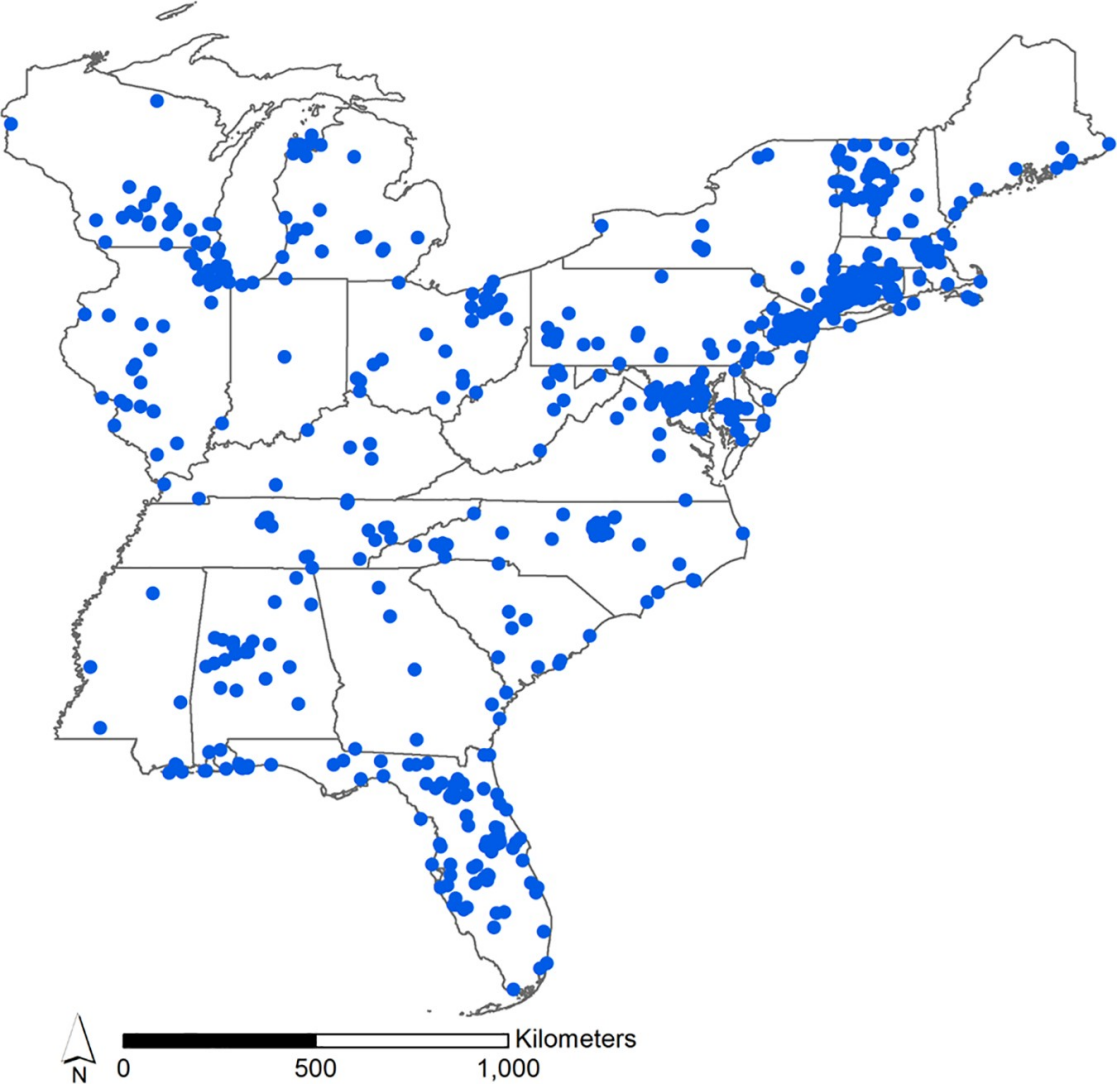
Locations of common ragweed occurrences throughout the eastern U.S.

We used geo-referenced locations of *Ambrosia artemisiifolia* (common ragweed) from the Global Biodiversity Information Facility (GBIF) online database (gbif.org). This dataset included over 3000 records of occurrence from 76 published datasets in the United States [22]. We then clipped the occurrence dataset with a shapefile of the eastern U.S. bounded by the Mississippi River on the west and the Atlantic Ocean on the east (Fig 1). We also removed occurrences that were outside of the contiguous U.S. resulting in 726 common ragweed occurrence records.

### Climate and bioclimatic data

Climate and bioclimatic data were downloaded from the WorldClim-Global Climate Data website (worldclim.org) (version 1.4). WorldClim provides global gridded climate data consisting of mean, maximum, and minimum temperature variables, precipitation variables, and bioclimatic variables with a spatial resolution of 30-arc seconds (about 1 km^2^) [23]. The historical dataset consists of interpolations of average monthly climate data from weather stations over the time period between 1960–1990.

Monthly climate data were also used to derive biologically relevant bioclimatic variables [23,24]. We included bioclimatic variables because they represent meaningful controls of plant growth and distribution [25]. We built and analyzed a number of ragweed distribution models to identify the most important predictor variables. We then examined correlations between those variables and selected four uncorrelated climate and bioclimatic variables, including precipitation seasonality, mean diurnal temperature range, August precipitation, and January maximum temperature. Precipitation seasonality is a measure of the variation in monthly precipitation totals over the course of the year [24]. Mean diurnal temperature range is the mean of the monthly temperature ranges (i.e., monthly maximum minus monthly minimum) [24]. The data were clipped by the same eastern U.S. extent as done with species occurrence data above.

The future climate data was downscaled from 13 individual global climate models (GCMs) from the Coupled Model Intercomparison Project Phase 5 (CMIP5) that were used in the Fifth Assessment of the Intergovernmental Panel on Climate Change (IPCC) (Table 1). We used downscaled future climate data from these 13 GCMs for two time periods: 2050s (averaged across 2041–2060) and 2070s (averaged across 2061–2080) and under two scenarios of future greenhouse gas emissions referred to as Representative Concentration Pathways (RCPs) 4.5 and 8.5. These greenhouse gas emissions scenarios apply socio-economic assumptions about future changes in global population, technological advances, and other factors that influence the amount of CO_2_ and other greenhouse gases emitted into the atmosphere as a result of human activities [26]. We used RCP 4.5 because it represents a low emissions scenario in which emissions stabilize by mid-century and decline thereafter, and RCP 8.5, which represents a high emissions scenario and assumes continued increases in greenhouse gas emissions until the end of the 21st century [26,27].

**Table 1:** Thirteen global climate models that were used in this study.

GCM	Institution
ACCESS1-0	Commonwealth Scientific and Industrial Research Organization (CSIRO) and Bureau of Meteorology (BOM), Australia
BCC-CSM1-1	Beijing Climate Center, China Meteorological Administration
CCSM4	US National Center for Atmospheric Research (NCAR)
CNRM-CM5	France National Centre for Meteorological Research
GFDL-CM3	NOAA/Geophysical Fluid Dynamic Laboratory (GFDL)
GISS-E2-R	National Aeronautics and Space Association Goddard Institute for Space Studies (NASA GISS)
HadGEM2-ES	UK Meteorological Office—Hadley Centre
INMCM4	Russian Institute for Numerical Mathematics (INM)
IPSL-CM5A-LR	Institute Pierre Simon Laplace (IPSL)
MIROC5	University of Tokyo, Japanese National Institute for Environmental Studies (NIES), and Japan Agency for Marine-Earth Science and Technology (JAMSTEC)
MPI-ESM-LR	Max Planck Institute (MPI) for Meteorology (low resolution)
MRI-CGCM3	Japanese Meteorological Research Institute (MRI)
NorESM1-M	Norwegian Climate Centre

### Species distribution model

We built a species distribution model for common ragweed using Maxent software [28]. In general, species distribution models identify a relationship between a species’ presence and a number of environmental or climate variables observed at those locations [29]. Once built, species’ distribution models then can be used to predict the suitability of a grid cell. This suitability is a function of the previously identified relationship for that given species and the environmental variables. Suitability then can be modeled for other locations or under different conditions (i.e., future climates). The Maxent software uses a machine-learning technique called maximum entropy modeling, which finds the distribution that best represents the data given all the available information [30,31]. Maxent is one of the better performing predictive modeling techniques and has been widely used for modeling species’ distributions [32].

We built our Maxent model using ragweed occurrence data and climate and bioclimatic variables to predict probability distributions across 3.3 million grid cells. We split our data set into two sets–a randomly selected 70% of the data for model training and 30% for model evaluation. To test the predictive performance of our model we resampled the test data set 500 times and report the area under the Receiver Operating Characteristic curve (AUC) [33] and the fraction of our study area that our model predicted present [30]. To model the future distribution of ragweed we used downscaled climate projections from thirteen individual GCMs for the 2050s and 2070s and two greenhouse gas emissions scenarios–RCP 8.5 and RCP 4.5.

## Results

**Fig 2:**
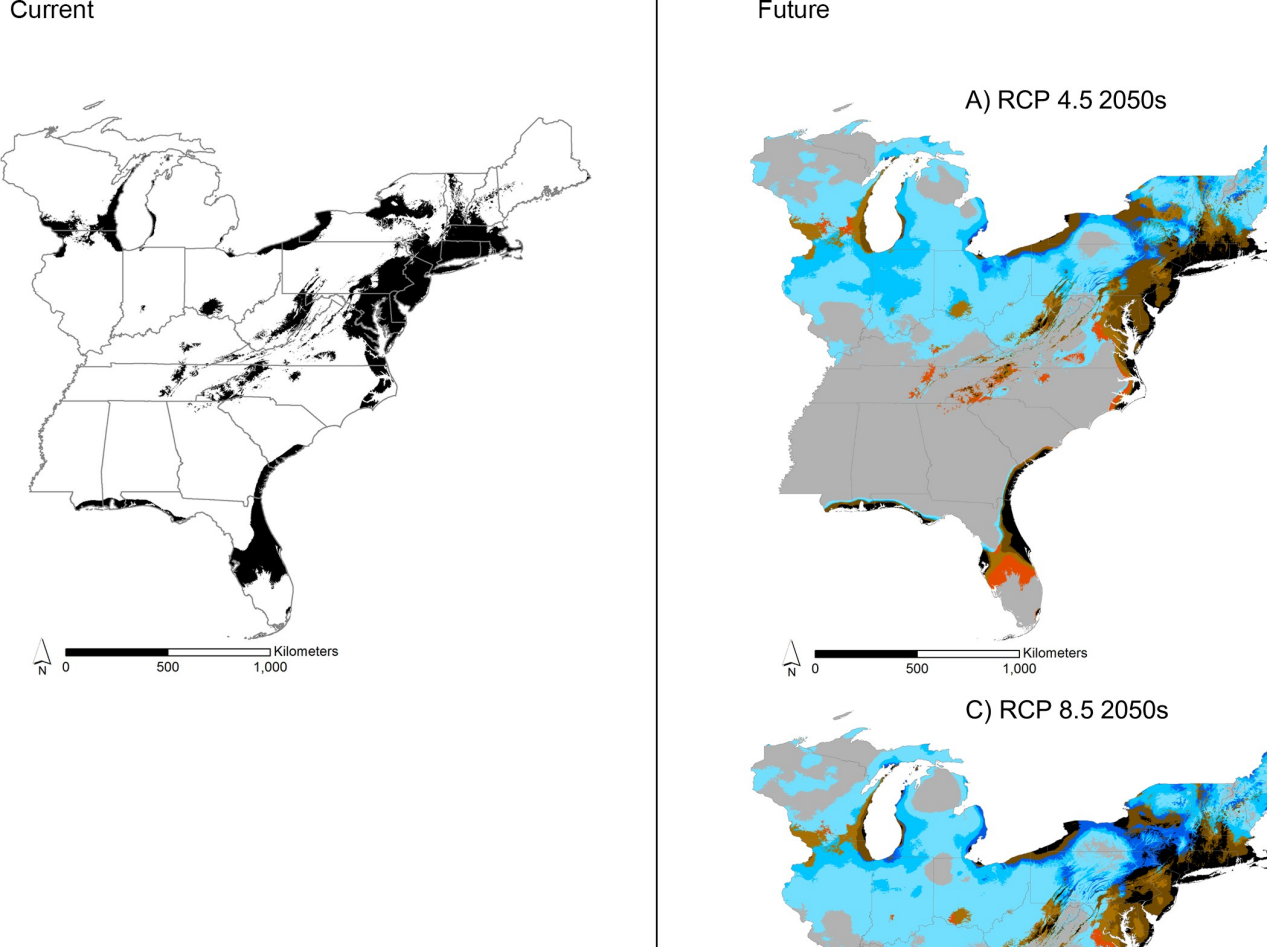
Predicted current and future presence (occurrence) of common ragweed across the eastern U.S. Left panel represents current predictions and the right panel represents the future distribution projections for RCP 4.5 and 8.5 and the 2050s and 2070s. The intensity of the colors represent agreement among the 13 global climate models.

**Fig 3:**
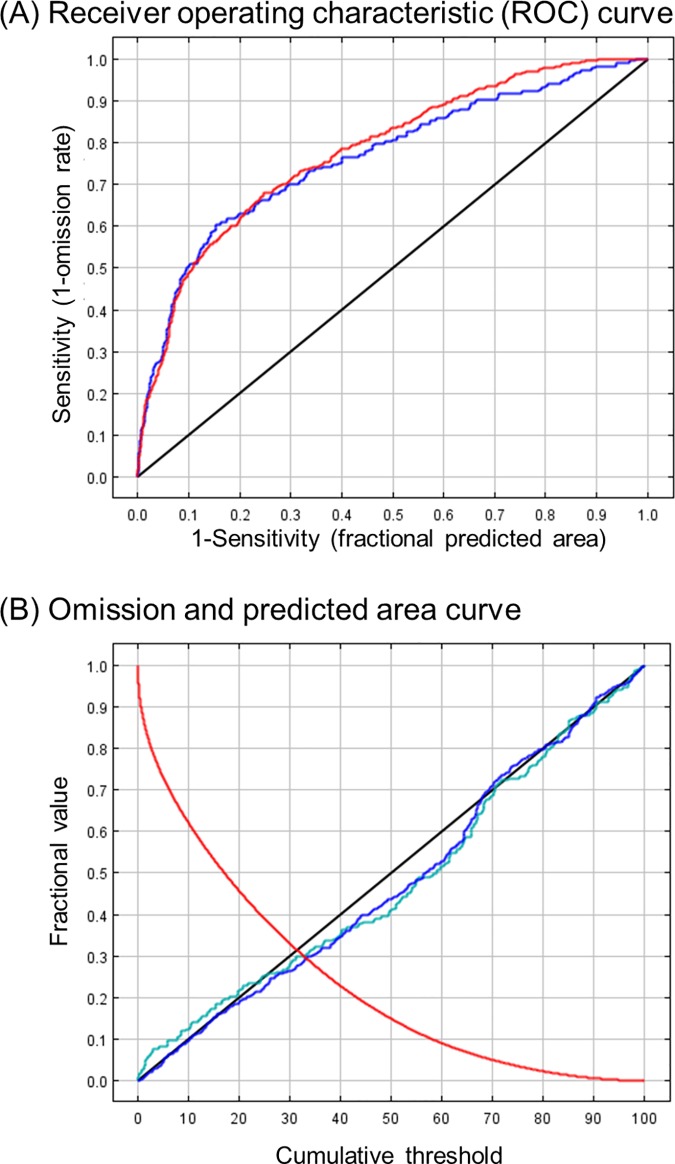
Model evaluation for common ragweed. (A) Receiver operating characteristic (ROC) curve. Red line is for the training data (AUC = 0.780), blue line is for the test data (AUC = 0.765), and black line is for a random set of predictions (AUC = 0.5). (B) Omission and predicted area curve. Red line is the fraction of background that is predicted, blue line is the omission rate for the training samples, teal line is the omission rate for the test samples, and black line is the predicted omission.

Our species distribution model was able to accurately predict 85% of ragweed’s presences across the study area (Fig 2, left panel). Analyzing the reserved test data set, our model had an AUC score of 0.765, and the omission rate–that is predicting no presence when one is there–was 0.396 (*p* < 0.001, one-tailed binomial test) (Fig 3). The occurrence data that we used did not include true absence information for common ragweed. Therefore, we calculated the fraction of the total study area that our model predicted present and compared this to a random prediction of presences with an AUC of 0.5 [30]. This technique uses pseudo-absences instead of true absences to calculate the percent of correctly predicted absences [34]. The fraction of the study area that our model predicted present was 0.154.

**Fig 4:**
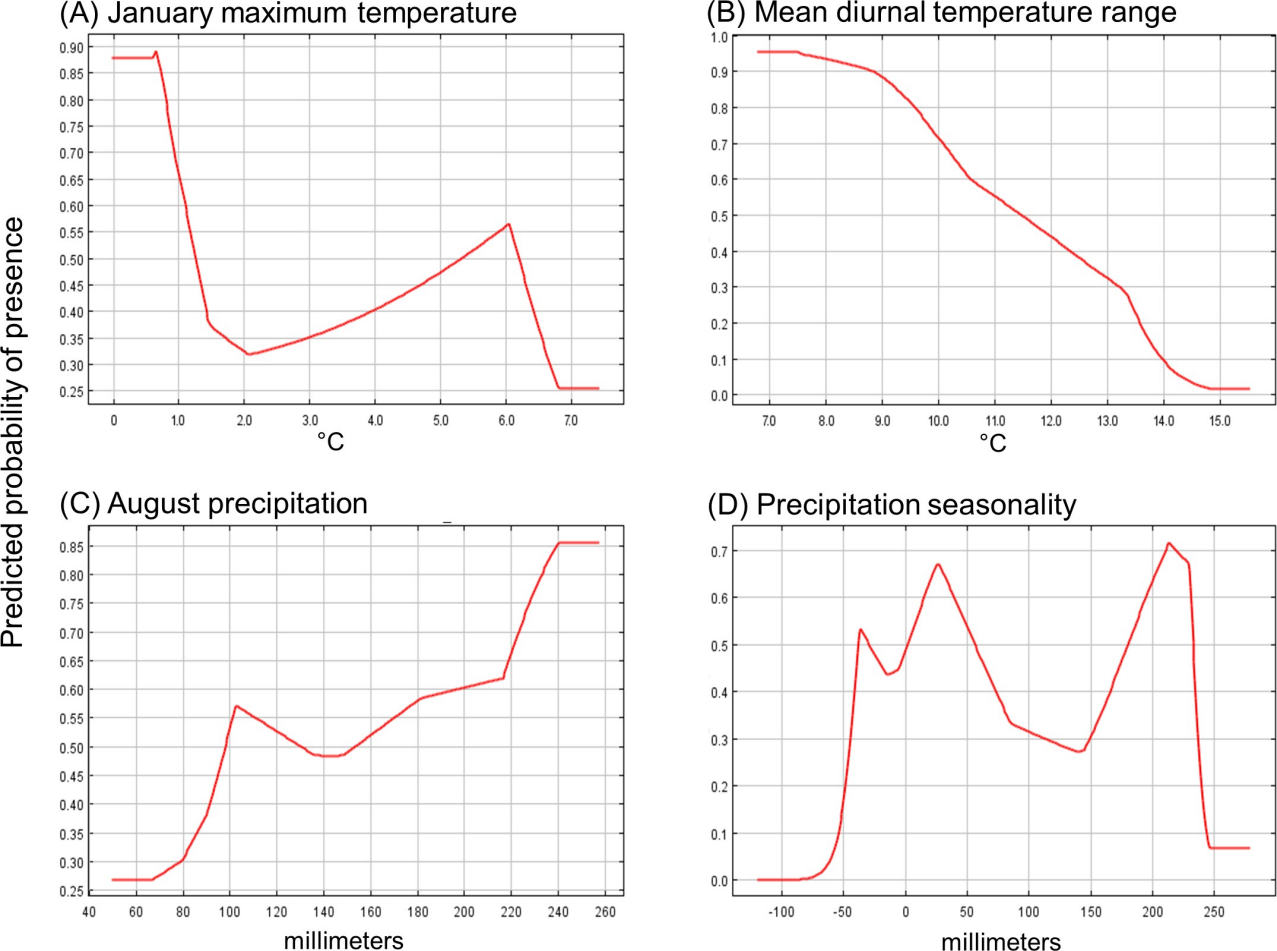
Response curves for the four climate variables used to predict common ragweed presence. (A) January maximum temperature, (B) mean diurnal temperature range, (C) August precipitation, and (D) precipitation seasonality. Temperature variables in plots A and B have been multiplied by a factor of 10. For example, 5 degrees C is represented by “50”.

We built the most parsimonious model possible while still maintaining a relatively high AUC score and predicting 85% of ragweed’s presences. We used four uncorrelated variables, including precipitation seasonality, mean diurnal temperature range, August precipitation, and January maximum temperature. Of these four variables, mean maximum temperature in January contributed the most to ragweed predictions, followed by precipitation seasonality, mean diurnal temperature range, and August precipitation. The response curve of how mean maximum temperature during January affects the predicted probability of ragweed occurrence (Fig 4A) shows a bimodal response with peaks below 1°C and 6°C. Predicted ragweed occurrence also had a negative relationship with mean diurnal temperature range (Fig 4B) and a positive relationship with August precipitation (Fig 4C). The relationship between predicted ragweed occurrence and precipitation seasonality was more complex–ragweed had a negative relationship in response to very low and very high precipitation seasonality and a positive relationship with moderate precipitation seasonality (Fig 4D).

The area of suitable climate space for common ragweed is projected to increase in the future but varies by greenhouse gas scenario and time period (Table 2). Interestingly, the area projected to be suitable for ragweed was greatest during the 2050s as compared to the 2070s under both greenhouse gas emissions scenarios. The higher scenario–RCP 8.5 –had larger increases of suitable area for ragweed compared to RCP 4.5 for the 2050s, but less of an increase for the 2070s compared to RCP 4.5. Because ragweed occurrence varies considerably by GCM, we used an ensemble to summarize all 13 models for each greenhouse gas scenario and time period (Fig 2). All future projections of suitable area for ragweed show substantial contraction in central Florida, southern Appalachian Mountains, and northeastern Virginia. Areas of potential ragweed expansion occur in the northern states with CGM model agreement highest in New York, Vermont, New Hampshire, and Maine. All future projections also agree that Massachusetts, Connecticut, Rhode Island, and the coastal areas of New Jersey, Delaware, Virginia, and North Carolina will remain suitable for ragweed during the current century.

**Table 2:** Future projected change in suitable area for common ragweed compared to the current distribution.

Scenario	Timeframe	Expansion	Contraction	Stable	Overall Change
		(Km^2^)	(Km^2^)	(Km^2^)	
RCP 4.5	2050s	838,175	258,609	83,165	+94%
	2070s	811,381	247,620	94,154	+92%
RCP 8.5	2050s	874,354	231,427	110,348	+120%
	2070s	800,113	275,262	66,513	+73%

There are also a number of differences among the four future projections. For instance, RCP 8.5 for the 2050s projects the largest amount of suitable climate space for ragweed, with the largest amount of expansion, smallest amount of contraction, and the most stable area. By contrast, RCP 8.5 for the 2070s projects the smallest amount of suitable climate space in the future and the most amount of overall change compared to ragweed’s current distribution (Table 2). Nevertheless, RCP 8.5 for the 2070s shows the largest amount of potential expansion and agreement among the 13 GCMs in northeast U.S.

## Discussion

Our results show that common ragweed is projected to have substantially more suitable climate space in the future across the eastern U.S. However, the vast majority of this increase is projected to occur by mid-century and may be moderated somewhat by the 2070s. This finding implies that common ragweed may be sensitive to climatic variability. For instance, we found that ragweed is positively correlated with increasing August precipitation, which was not surprising. However, ragweed was also negatively correlated to very low or very high annual precipitation variability, indicating a general sensitivity to precipitation extremes. Ragweed’s sensitivity to water stress has been identified by others [19,20]. In Europe, where ragweed is an exotic invasive and has not yet maximized its full niche, it appears to be limited by strong summer drought [19]. Ragweed is also negatively correlated to the mean of monthly temperature ranges indicating that ragweed is sensitive to temperatures extremes, a finding supported by other studies [19,20]. Our results also support the notion that ragweed is intolerant to frost [20,35].

Species distribution models and their projections of newly suitable habitat can be useful for land management planning [36,37]. Our results identify a number of locations that ragweed is not currently present but may expand into in the future and thus become a new or increased health concern. For example, metropolitan areas in the Northeast, such as Albany, New York, Montpelier, Vermont, Concord, New Hampshire, and Augusta, Maine are all at increased risk of ragweed expansion in the next 30 years. An expansion of ragweed at its northern margins of its current distribution is generally consistent with European studies [18,19,21,38]. Therefore, Northeast metropolitan areas may be well advised to start monitoring for ragweed presence and potentially increase eradication efforts by local weed control boards. By contrast, some areas in southern Vermont and New Hampshire and parts of Massachusetts may become less suitable for ragweed by the end of the century. These areas may provide opportunities to displace ragweed with later-successional species [39]. It is important to recognize that ragweed is adapted to a diversity of habitats and tends to grow competitively on disturbed soils [10]. Therefore, urban development and planning efforts may consider minimizing the exposure of disturbed sites by requiring adequate soil coverage with other vegetation, or timing soil disturbance to occur in winter and spring, while ragweed is not dispersing.

We modeled ragweed distribution using climate predictor variables only. Alternative modeling approaches could also include other factors, such as dispersal, land-use change, CO_2_ concentrations, and geographic and ecotypic variation among ragweed populations. Others have also developed mechanistic modeling approaches to predict ragweed distribution [20,38] and cite that climate envelope modeling may underestimate the true potential range of invasive species [21,40]. We agree. Integrating mechanistic and empirical modeling approaches may improve the predictive power and certainty associated with future projections [41]. Predictive models could also be parameterized and restrained with known dispersal abilities, as has been done with common ragweed in Europe [42].

Land use and disturbance history are also important, known drivers of ragweed distribution [16]. We did not include land use in our final model because regional projections of future land use change were not available. However, we did explore the relative influence of land use in determining ragweed’s current distribution with earlier exploratory models. Specifically, we included three land cover variables–National Land Cover data, tree canopy cover data, and a dataset of impervious surfaces [43]–along with our climate variables to predict ragweed occurrence. The dataset of impervious surfaces was an important predictor variable in early exploratory model runs. Therefore, future research will further examine the role of land use and how projected future changes in both land use and climate change may impact ragweed occurrence. We also recognize that there is geographic and ecotypic variation among ragweed populations [17,20,44], but to our knowledge incorporating this variation into predictive models for the U.S. has not been done.

Another factor that influences the growth and potentially the establishment of ragweed is the greenhouse gas CO_2_. Specifically, common ragweed has been shown to respond positively to elevated CO_2_ [13,14,45], and global CO_2_ concentrations are increasing [46]. Although we did not include CO_2_ concentrations in our model, future research could also integrate this factor into future projections of ragweed growth and distribution. There are many facets of modeling ragweed that could be explored. Nevertheless, we have demonstrated that a relatively simple climate niche model can predict its current distribution. Although not intended for fine-scale mapping of ragweed occurrence, our model can be used to highlight key climatic drivers and inform management actions.
